# Ozanimod for Treatment of Relapsing-Remitting Multiple Sclerosis in Adults: A Systematic Review and Meta-Analysis of Randomized Controlled Trials

**DOI:** 10.3389/fphar.2020.589146

**Published:** 2020-11-20

**Authors:** Yue Sun, Yanbo Yang, Zilan Wang, Fan Jiang, Zhouqing Chen, Zhong Wang

**Affiliations:** ^1^ Department of Neurosurgery and Brain and Nerve Research Laboratory, The First Affiliated Hospital of Soochow University, Suzhou, China; ^2^ School of Biology and Basic Medical Science, Soochow University, Suzhou, China; ^3^ First Clinical Medical School of Soochow University, Suzhou, China

**Keywords:** meta-analysis, new or enlarging T2 lesions, gadolinium-enhancing lesions, ozanimod, relapsing-remitting multiple sclerosis, annualized relapse rate

## Abstract

**Background:** Ozanimod has been approved for use in the treatment of relapsing forms of multiple sclerosis by the United States FDA. As a novel, orally available sphingosine 1-phosphate receptor modulator, ozanimod selectively binds to S1P1 and S1P5 receptor with high affinity, minimizing safety concerns caused by S1P_3_ receptor activation.

**Methods:** e systematically searched PUBMED, EMBASE database, and Cochrane Library database to identify randomized controlled trials (RCTs) from inception to June 28, 2020. Trials were considered eligible if they 1) were randomized clinical trials (RCTs); 2) enrolled adult participants diagnosed with Relapsing-remitting MS; 3) compared ozanimod with placebo or any other approved DMDs that evaluated in phase III or phase II clinical trials; 4) enrolled over 100 participants; 5) provided any available information for predefined primary or secondary outcomes.

**Results:** 2917 participants from three high-quality, multi-centered randomized clinical trials were pooled in our analysis. We found that using ozanimod was significantly associated with the reduction of the annualized relapse rate during the treatment period (RR, −0.10 [95% CI, −0.15, −0.06]). Also, the decreased number of gadolinium-enhancing lesions at the end of the trial was relative to the treatment of ozanimod (ozanimod, 0.29; control, 0.65; RR, −0.20 [95% CI, −0.34, −0.06]). Compared with patients in the control group, the number of new or enlarging T2 lesions over the treatment period decreased in patients treated with ozanimod (ozanimod, 1.82; control, 3.55; RR, −1.12 [95% CI, −1.52, −0.71]). As to the safety endpoints, patients in the ozanimod group reported a lower rate of adverse events (ozanimod, 66.03%; control, 77.07%; RR, 0.64 [95% CI, 0.43, 0.95]). Similar incidence of infection-related TEAEs was found across treatment groups (nasopharyngitis: ozanimod, 11.19%; control, 9.83%; RR, 1.10 [95% CI, 0.77–1.57]; urinary-tract infection: ozanimod, 3.81%; control, 2.97%; RR, 1.29 [95% CI, 0.83–2.00]). No case of macular edema was noted as well as second-degree, type 2, or third-degree atrioventricular block. As for the subgroup analysis, compared with 0.5 mg ozanimod, 1 mg ozanimod is related with a significant reduction of the annualized relapse rate during the treatment period (1 mg ozanimod, 0.18; 0.5 mg ozanimod, 0.24; RR, 0.05 [95% CI, 0.01, 0.09])and a decreased number of new or enlarging T2 lesions over the treatment period (1 mg ozanimod,1.58; 0.5 mg ozanimod, 2.05; RR, 0.49 [95% CI, 0.19, 0.79]). No significant difference in causing adverse events between 1 and 0.5 mg was found.

**Conclusions:** Our meta-analysis found that, with favorable safety performance, the use of ozanimod as a treatment of relapsing-remitting multiple sclerosis in adults was associated with a significant reduction of the annualized relapse rate during the treatment period, decreased number of gadolinium-enhancing lesions at the end of the trial, and lowered number of new or enlarging T2 lesions over the treatment period. Ozanimod 1 mg outperformed 0.5 mg dose in efficacy without increasing the risk of adverse events.

## Introduction

Multiple Sclerosis (MS) is a chronic, demyelinating, and neurodegenerative disease of the central nervous system (CNS), mainly affecting young adults (Dutta & Trapp, 2014). Relapsing-remitting MS(RRMS) is the most prevalent presented by nearly 85% of individuals with MS (European Medicines Agency, 2015). The immunopathogenesis of MS is considered to be related to self-tolerance toward myelin and other CNS antigens (Hafler et al., 2005). Bidirectional interactions between immune cells in the periphery such as T cells, B cells, and resident cells of the CNS such as microglia and astrocytes are found to play an essential role in the pathophysiology of MS (Li et al., 2018). Secreting inflammatory mediators play a pivotal role in the pathogenesis of MS. CNS-resident proinflammatory cells can be activated by these inflammatory mediators, together with peripheral immune cells that were recruited and stimulated by secreting inflammatory mediators, can cause an inflammatory response to self-antigens, and lead to neuronal demyelination (Filippi et al., 2018). Based on these findings, more tailored therapeutic approaches and clinical trials have developed rapidly.

Sphingosine-1-receptor modulators are one of the targets for disease-modifying therapies (DMTs) (Faissner & Gold, 2019). Bonding to the S1P receptors on lymphocytes that induce internalization and degradation of S1P receptors, sphingosine 1-phosphate (S1P) receptor modulators prevent lymphocytes to egress from the lymphoid tissue (Scott et al., 2016). As the first approved S1P receptor for the treatment of RRMS, clinical trials have demonstrated that the use of fingolimod was related to decreased relapse rate, and the number of gadolinium-enhancing lesions, new or enlarging T2 lesions, brain volume loss on MRI, and impedes disability progression (Doggrell, 2010; Calabresi et al., 2014). However, as a non-selective S1P receptor, fingolimod binds to multiple subtypes of S1P, potentially lead to significantly increased adverse events such as bradycardia, macula edema, and dyspnoea (Sørensen, 2016).

Ozanimod is an oral selective S1P receptor modulator that selectively targets the receptor subtypes S1PR_1_ and S1PR_5_. Ozanimod was demonstrated to decrease the absolute number of lymphocytes, reducing lymphocyte subsets that express cytokine receptor 7 (Tran et al., 2017). Also, the neuroprotective potential of ozanimod was revealed by the decrease of plasma neurofilament light chain (Taylor Meadows et al., 2017; Harris et al., 2019). Several phase III clinical trials have been performed to evaluate the efficacy and safety of ozanimod for the treatment of RRMS with positive results. Subsequently, ozanimod was approved for the treatment of RRMS as 0.25, 0.5, and 1 mg ozanimod HCl (Lamb, 2020). However, until now, no systematic approach has been performed to evaluate safety and efficacy endpoints across different doses of ozanimod. Therefore, we conducted a meta-analysis to evaluate the efficacy and tolerability of ozanimod for the treatment of RRMS.

## Method

### Search Strategy

PUBMED, EMBASE database, and Cochrane Library database were systematically searched to identify randomized controlled trials (RCTs) from inception to June 28, 2020. Search terms include ozanimod, multiple sclerosis, randomized clinical trials. Vocabulary and syntax were adjusted across databases. We also found other references by manually searching bibliographies of correlative articles. Two investigators independently screened the list of articles from search results to ensure all relevant studies would be enrolled in our study. Any disagreement was settled through discussion.

### Inclusion and Exclusion Criteria

Trials were considered eligible if they (Dutta & Trapp, 2014) were randomized clinical trials (RCTs); (European Medicines Agency, 2015) enrolled adult participants diagnosed with RRMS (Hafler et al., 2005); compared ozanimod with placebo or any other approved DMDs that evaluated in phase III or phase II clinical trials (Li et al., 2018); enrolled over 100 participants (Filippi et al., 2018); provided any available information for predefined primary or secondary outcomes.

### Study Selection and Data Collection

In the ﬁrst phase of screening, the titles and abstracts of all identiﬁed citations were screened by two independent reviewers. In the second phase of screening, full manuscripts were retrieved and screened by two independent reviewers on the basis of our pre-determined information consisting of the patient population, intervention, comparison, outcomes, and study design of interest. All controversies were settled by consensus. For evaluating the biases of included RCTs, we used criteria of the Cochrane collaboration, including selection bias, performance bias, detection bias, attrition bias, reporting bias, and other bias. As only three available trails are included, publication bias is not evaluated. The risk of bias in each study was studied and plotted by using the Review Manager 5.3 software.

### Outcomes

The pre-determined co-primary efficacy outcomes include: the annualized relapse rate (ARR) during the treatment period; secondary efficacy endpoints include the number of gadolinium-enhancing brain lesions at the end of clinical trial and the number of new or enlarging T2 lesions over the treatment period; safety endpoints include any reported death and treatment-emergent adverse events in included trails.

### Subgroup Analysis

Subgroup analyses were performed to answer speciﬁc questions, such as the effects of types of interventions (different dosages). I^2^ is used to address heterogenicity between studies.

### Statistical Analysis

The outcomes involved continuous and count data. We used the weighted mean difference with 95% CIs for continuous data and the rate ratio with 95% CI for count data. The heterogeneity between the included studies was evaluated with I^2^ and *p* value. Data will be considered to show significant heterogeneity when the I^2^ >50% or *p*-value <0.05. A random-effects model was used. All the analysis was conducted with Review Manager 5.3 software.

## Results

### Baseline Characteristic

**FIGURE 1:**
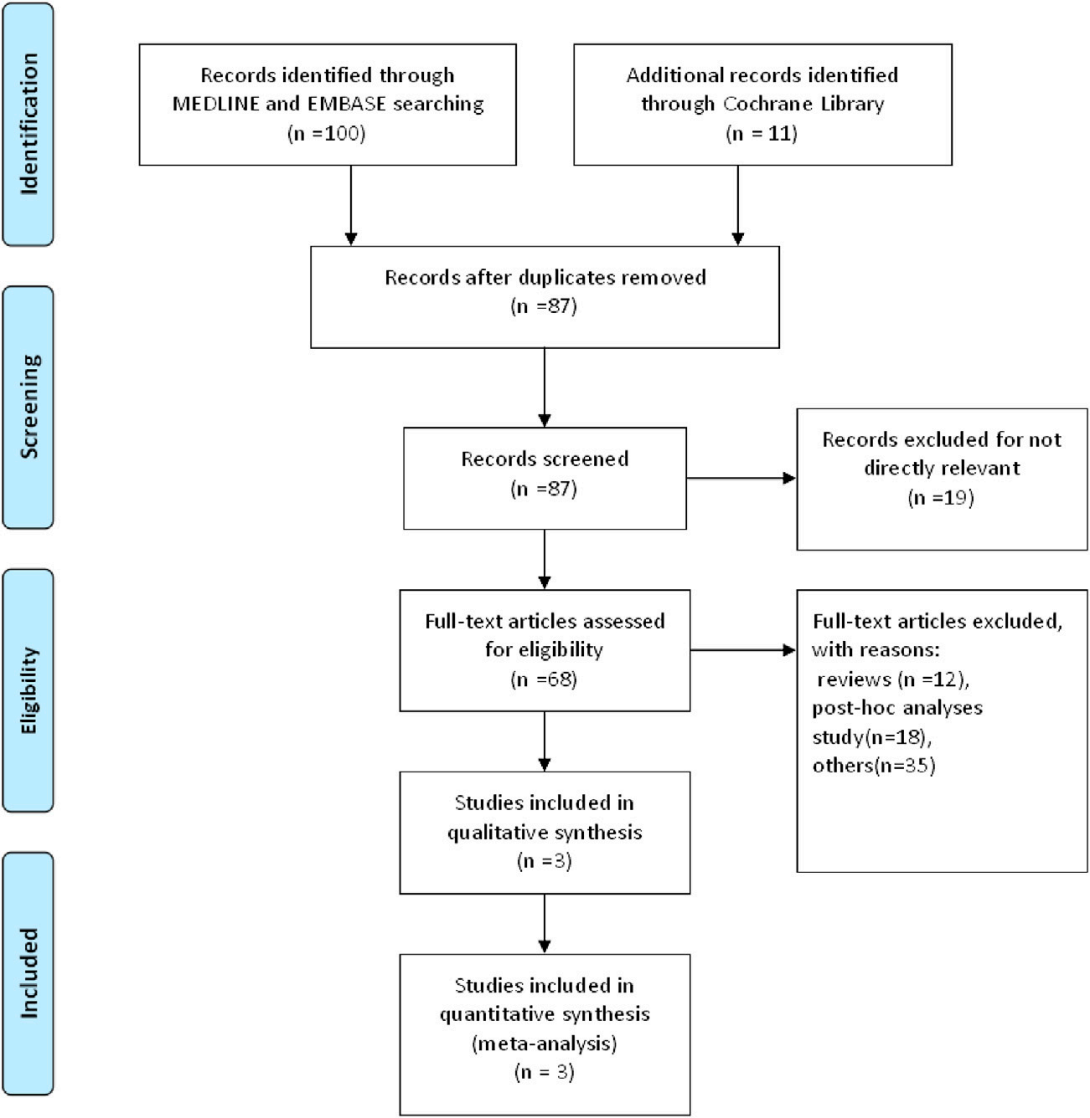
PRISMA Flow Diagram of the study inclusion process.

**FIGURE 2:**
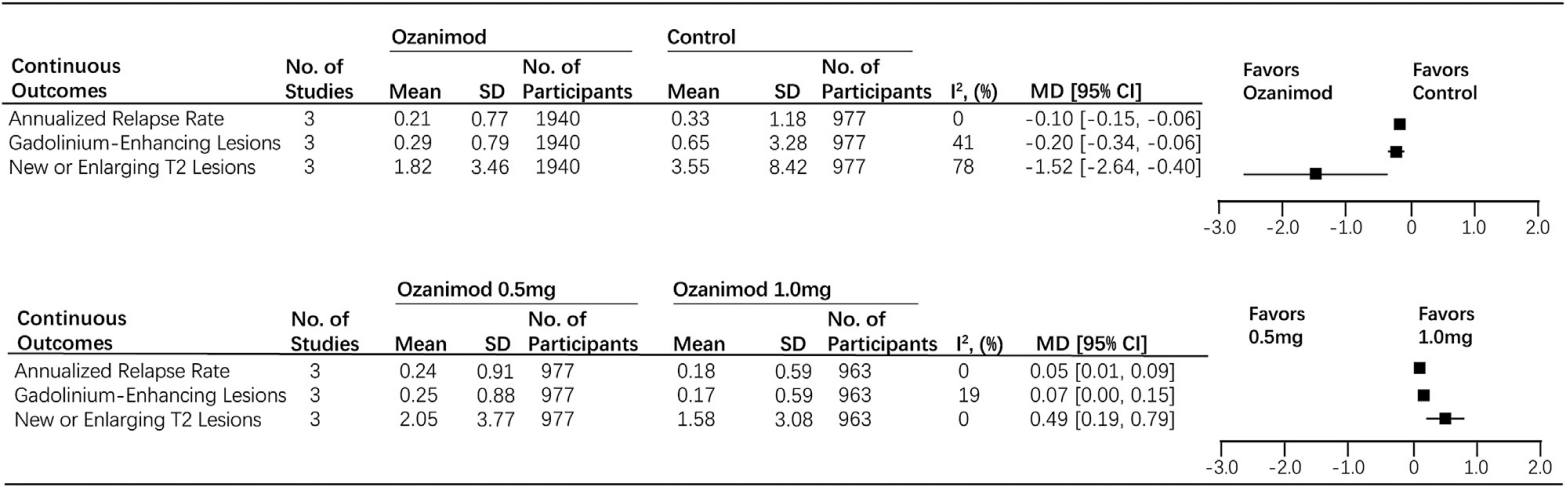
Risk of bias.

Overall, 2917 patients with RRMS from three multi-centered randomized clinical trials were pooled in our study (Cohen et al., 2016; Cohen et al., 2019; Comi et al., 2019) (Table 1). According to the baseline characteristics in each study integrated, 1957 (67.1%) were female, 2558 (87.7%) had a white ethnicity. The expanded disability status scale score (EDSS) at baseline was 2.58. Patients had 1.31 and 1.76 times of relapses in the previous 12 and 24 months, respectively. Dosage subgroups included in our study were 0.5 and 1 mg. The study selection process was plotted in Figure 1. The risk of bias for each study was plotted in Figure 2.

**TABLE 1:** Baseline characteristics of included studies.

Study baseline characteristics Treatment arms	Cohen et al., 2016			Cohen et al., 2019			Comi et al., 2019		
	Control (n = 88)	Ozanimod 0.5 mg (n = 87)	Ozanimod 1 mg (n = 83)	Control (n = 441)	Ozanimod 0.5 mg (n = 439)	Ozanimod 1 mg (n = 433)	Control (n = 448)	Ozanimod 0.5 mg (n = 451)	Ozanimod 1 mgg (n = 447)
Age(SD)	39.0 (8.7)	38.1 (9.2)	38.4 (9.8)	35.1 (9.1)	35.4 (8.8)	36.0 (8.9)	35.9 (9.1)	36.0 (9.4)	34.8 (9.2)
Female sex,%	62 (70)	60 (69)	59 (71)	304 (68.9)	287 (65.4)	291 (67.2)	300 (67.0)	311 (69.0)	283 (63.3)
White ethnic origin,%	87 (99)	84 (97)	83 (100)	432 (98.0)	431 (98.2)	428 (98.8)	447 (99.8)	447 (99.1)	446 (99.8)
Relapses in 12 months before screening	1.3 (0.6)	1.5 (1.2)	1.3 (0.7)	1.3 (0.58)	1.4 (0.64)	1.3 (0.56)	1.3 (0.6)	1.3 (0.6)	1.3 (0.6)
Relapses in 24 months before screening	1.8 (1.0)	2.0 (1.8)	1.9 (1.1)	1.8 (0.86)	1.8 (0.90)	1.7 (0.82)	1.7 (0.8)	1.7 (0.8)	1.8 (0.9))
Gadolinium -enhancing lesions at baseline volume, cm	—	—	—	0.25 (0.62)	0.23 (0.53)	0.21 (0.53)	0.18 (0.46)	0.16 (0.41)	0.20 (0.54)
EDSS score	2.9 (1.3)	2.9 (1.3)	2.9 (1.2)	2.5 (1.16)	2.5 (1.17)	2.6 (1.15)	2.6 (1.1)	2.7 (1.1)	2.6 (1.2)

EDSS: expanded disability status scale score.

### Efficacy Endpoints Comparing the Ozanimod Group With the Control Group

**FIGURE 3:**
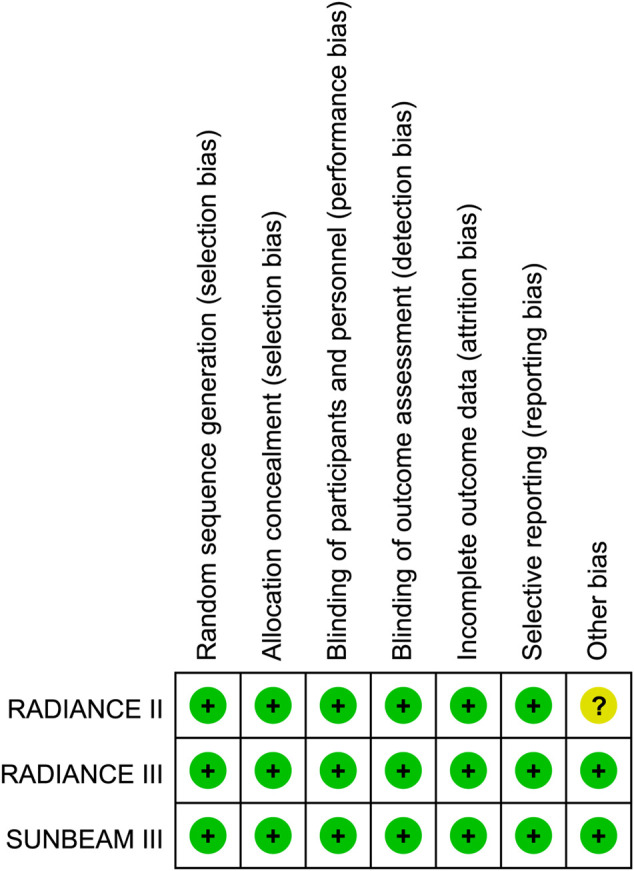
Efficacy endpoints for ozanimod compared with control and 0.5 mg ozanimod compared with 1 mg dose.

For the primary efficacy endpoint, the use of ozanimod is significantly relative to the reduction of the annualized relapse rate during the treatment period (ozanimod, 0.21; control, 0.33; RR, −0.10 [95% CI, −0.15, −0.06], *p* < 0.0001), no significant heterogeneity was found (I^2^ = 0%). For the secondary efficacy endpoints, patients treated with ozanimod had less number of gadolinium-enhancing lesions at the end of the trial compared with patients in the control group (ozanimod, 0.29; control, 0.65; RR, −0.20 [95% CI, −0.34, −0.06], *p* = 0.006), with a moderate heterogeneity (I^2^ = 41%). Also, in comparison with patients in the control group, the number of new or enlarging T2 lesions over the treatment period in the ozanimod group was decreased (ozanimod, 1.82; control, 3.55; RR, −1.52 [95% CI, −2.64, −0.40], *p* = 0.008, I^2^ = 78%) (Figure 3). Forest plots of mentioned outcomes are listed in Supplementary Material 1.

### Safety Endpoints Comparing the Ozanimod Group With the Control Group

**FIGURE 4:**
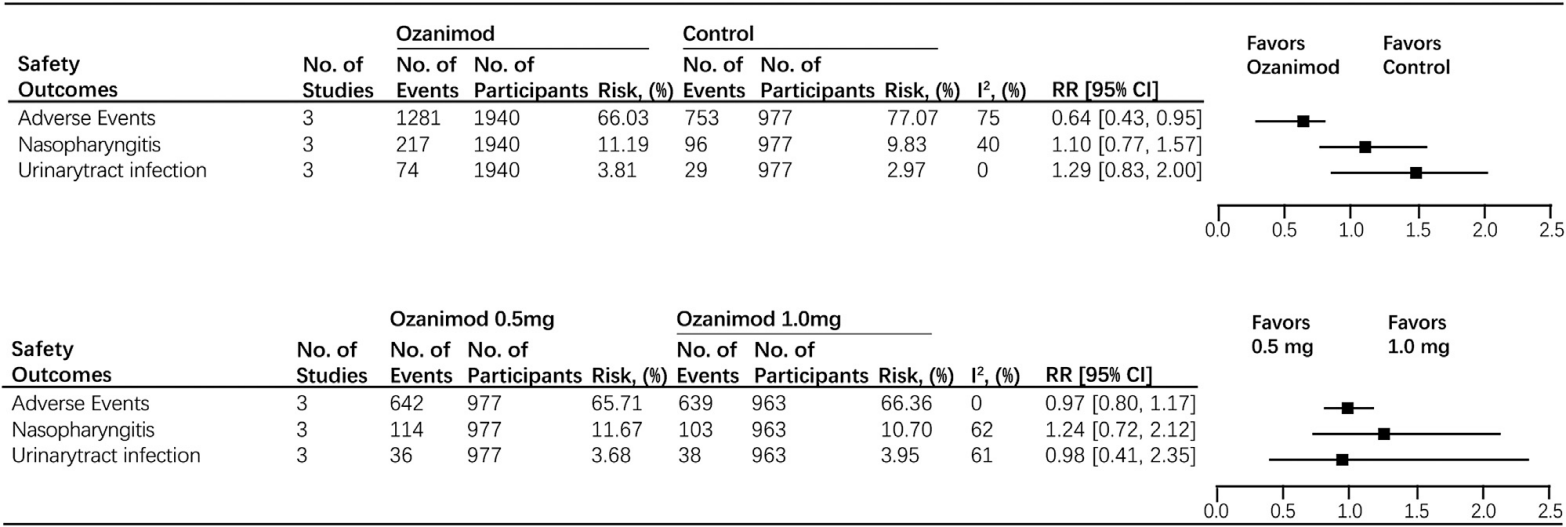
Safety outcomes for ozanimod compared with control and 0.5 mg ozanimod compared with 1 mg dose.

Most of the treatment-emergent adverse events (TEAES) were mild or moderate in severity. Nasopharyngitis (15.39%) is the most commonly reported treatment-emergent adverse event. It is demonstrated that the use of ozanimod is associated with the decreased occurrence of adverse events (ozanimod, 66.03%; control, 77.07%; RR, 0.64 [95% CI, 0.43, 0.95], *p* = 0.03, I^2^ = 75%). The incidence of infection-related TEAEs was similar across treatment groups (nasopharyngitis: ozanimod, 11.19%; control, 9.83%; RR, 1.10 [95% CI, 0.77–1.57]; urinary-tract infection: ozanimod, 3.81%; control, 2.97%; RR, 1.29 [95% CI, 0.83–2.00]) (Figure 4). No case of macular edema (ME) was noted as well as second-degree, type 2, or third-degree atrioventricular block. Forest plots of mentioned outcomes are listed in Supplementary Material 1.

### Efficacy Endpoints Comparing Ozanimod 0.5mg With Ozanimod 1.0mg

The baseline characteristics are balanced in the 0.5 mg group and 1.0 mg group. For the primary efficacy endpoint, patients treated with 1 mg ozanimod had a better performance in reducing ARR compared with patients in 0.5 mg group (1 mg ozanimod, 0.18; 0.5 mg ozanimod, 0.24; RR, 0.05 [95% CI, 0.01, 0.09], *p* = 0.007). As for the secondary efficacy endpoints, the number of new or enlarging T2 lesions over the treatment period was lower in 1.0 mg group in comparison with the 0.5 mg one (1 mg ozanimod, 1.58; 0.5 mg ozanimod, 2.05; RR, 0.49 [95% CI, 0.19, 0.79], *p* = 0.001). However, patients treated with different dosages showed no significant disparity in decreasing the number of gadolinium-enhancing lesions at the end of the trial (1 mg ozanimod, 0.17; 0.5 mg ozanimod, 0.25; RR, 0.07 [95% CI, 0.00, 0.15], *p* = 0.06) (Figure 3). Heterogeneity was low for all outcomes (I^2^ range: 0–20%). Forest plots of mentioned outcomes are listed in Supplementtary Material 1.

### Safety Endpoints Comparing Ozanimod 0.5 mg With Ozanimod 1.0 mg

There was no significant difference in causing adverse events between two dosages (1 mg ozanimod, 66.36%; 0.5 mg ozanimod, 65.71%; RR, 0.97 [95% CI, 0.80, 1.17], *p* = 0.76). No heterogeneity was found (I^2^ = 0). In addition, no disparity was found between 0.5 and 1.0 mg dosage subgroups for both nasopharyngitis and urinary-tract infection (nasopharyngitis:1 mg ozanimod, 10.70%; 0.5 mg ozanimod, 11.43%; RR, 1.24 [95% CI,0.72, 2.12], *p* = 0.44; urinary-tract infection:1mg ozanimod, 3.95%; 0.5 mg ozanimod, 3.68%; RR, 0.98 [95%CI, 0.41, 2.35], *p* = 0.76). Significant heterogeneity was found for risk of patients developing nasopharyngitis (I^2^ = 62%) and urinary-tract infection (I^2^ = 61%) (Figure 4). Forest plots of mentioned outcomes are listed in Supplementtary Material 1.

## Discussion

Our study is the first meta-analysis of the clinical use of ozanimod for treating RRMS, we pooled up 2,917 participants from three high-quality, multi-centered randomized clinical trials, concluded that ozanimod was associated with a decreased annualized relapse rate for patients with RRMS. Also, the number of gadolinium-enhancing lesions at the end of the trial and new or enlarging T2 lesions over the treatment period were decreased in patients treated with ozanimod. As for the safety outcomes, compared with the control group, the occurrence of adverse events decreased in the ozanimod group with a similar incidence of infection-related TEAEs. In addition, ozanimod use is not associated with increased risk for developing macular edema, and second-degree, type 2, or third-degree atrioventricular block. As for the dose-response relationship, we found 1 mg ozanimod outperformed 0.5 mg ozanimod measured by ARR and the number of new or enlarging T2 lesions over the treatment period. Subsequently, no significant differences were found between these two dosage regimes for adverse events, including nasopharyngitis and urinary-tract infection. The heterogeneity level for the above-mentioned findings was low, indicating a high level of clinical evidence.

As an agonist of S1P_1_ and S1P_5_ with a more than ten-thousand-fold selectivity for S1P_1_ over S1P_2,3,4_ receptors, ozanimod minimizes safety concerns caused by S1P_3_ receptor activation, outperforming fingolimod which is a non-selective S1P receptor modulator (Cree et al., 2018; Gross et al., 2018; Swallow et al., 2020). Although atrial myocytes, cardiac symptoms after first administration could still occur, and the recommended titration should be followed, less extended first-dose monitoring was needed for patients treated with ozanimod (Swallow et al., 2020; ZEPOSIA, 2020). Also, compared with fingolimod, ozanimod was associated with a lower risk of other outcomes assessed currently, while being comparable to reduce ARR and impede disability progression (Swallow et al., 2020). Our study found 1 mg ozanimod outperforms 0.5 mg in reducing ARR and decreasing the number of new or enlarging T2 lesions over the treatment period, which can be explained by the fact that ozanimod exhibited linear pharmacokinetics with dose ranging from 0.3 to 3 mg (Tran et al., 2017). Currently, immunosuppressant or immunomodulatory including interferonβ-1a, interferonβ-1b (inhibits the activation of T cell and reduce the penetration of inflammatory cells through the blood-brain barrier), glatiramer acetate (activates Th2 cell and promotes the production of anti-inflammatory cytokines), teriflunomide (inhibits the proliferation of lymphocyte), and dimethyl fumarate (activates Nrf2) are used to treat RRMS (Katsara & Apostolopoulos, 2018). According to a network meta-analysis and number-needed-to-treat analyses of ozanimod, ozanimod has greater efficacy on the ARR and favorable number-needed-to-treat analyses outcomes, compared with placebo and other commonly used first-line DMTS (Kumar et al., 2019; Tencer et al., 2019).

Previous studies discovered that using ozanimod was related to the reduction of brain volume loss, cortical gray matter volume loss, and thalamic volume loss (Arnold et al., 2019). The correlations between BVL and disability/cognitive impairment suggested ozanimod may be beneficial in impeding longer-term disease worsening in RRMS patients (Arnold et al., 2017). The long-term trial has concluded that ozanimod could improve cognitive processing speed sustainably during an 18-months follow up (DeLuca et al., 2019). Up to now, an open-label extension (OLE) study has shown that ozanimod was generally well tolerated with no new safety concerns raised in the long-term (Steinman et al., 2019). In addition, as for pregnant women during the first trimester, ozanimod showed no sign of increasing the risk of fetal abnormalities or adverse pregnancy outcomes in a limited program, shedding light on the treatment during pregnancy (Campagnolo et al., 2018). It is known that RRMS often converts to a secondary-progressive disease course (SPMS), which leads to loss of ambulation because of a slow and steady accumulation of disability (Weinshenker et al., 1989), while there have no trials in progressive patients treated with ozanimod to test its efficacy. However, targeting the same receptors as siponimod, which is effective in SPMS, ozanimod is an auspicious medication for progression (Kappos et al., 2018). More clinical trials are needed.

## Limitation

Despite including three multi-centered randomized trials that have a low risk for bias, our study is still limited by the predominately white ethnicity. Also, only single and short-term trials were investigated, making it hard to figure out the effect of ozanimod in the long run and in cooperative ways.

## Conclusion

Ozanimod was associated with cutting down annualized relapse rate, the number of gadolinium-enhancing lesions at the end of the trial, and new or enlarging T2 lesions over the treatment period. Macular edema and cardiac symptoms as second-degree, type 2, or third-degree atrioventricular block are not directly related to the treatment of ozanimod. 1 mg ozanimod performs better than 0.5 mg with the same risk of developing adverse events.

## Author Contributions

ZC was the principal investigator. YS was the main contributor to design, statistical analysis, and writing the first manuscript. YY was responsible for revising the manuscript, checking the collected data, and validating of the included studies. Data collection, plotting, and editing analysis tables and graphs were allocated to other authors.

## Funding

This work was supported by the Suzhou Health Talents Training Project (GSWS2019002).

## Conflict of Interest

The authors declare that the research was conducted in the absence of any commercial or financial relationships that could be construed as a potential conflict of interest.
