# Supplementary material for: Ozanimod for Treatment of Relapsing-Remitting Multiple Sclerosis in Adults: A Systematic Review and Meta-Analysis of Randomized Controlled Trials
Source: Front Pharmacol. 2020 Nov 20;11:589146. doi: 10.3389/fphar.2020.589146 (PMC7919188; doi:10.3389/fphar.2020.589146)

Figure 1. Forest plot of annualized relapse rate during the treatment period ozanimod versus control; CI, confidence interval


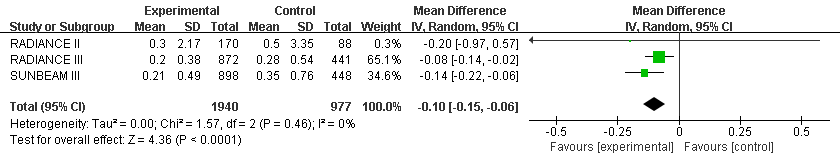


Figure 2. Forest plot of the number of gadolinium-enhancing lesions at the end of the trial ozanimod versus control; CI, confidence interval


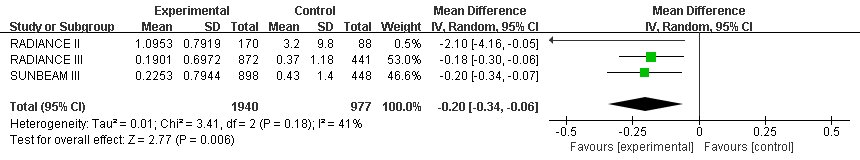


Figure 3. Forest plot of the number of new or enlarging T2 lesions over the treatment period ozanimod versus control; CI, confidence interval


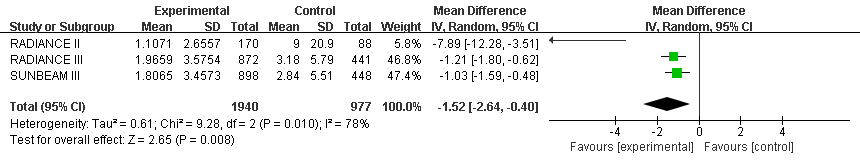


Figure 4. Forest plot of the treatment related adverse events ozanimod versus control; CI, confidence interval


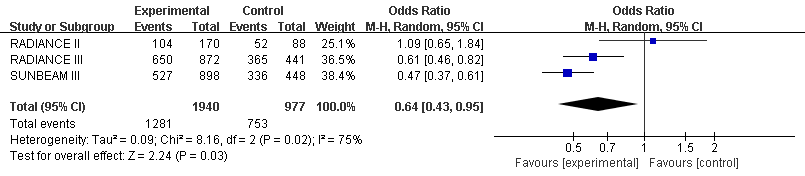


|  |
| --- |

Figure 5. Forest plot of treatment related nasopharyngitis ozanimod versus control; CI, confidence interval


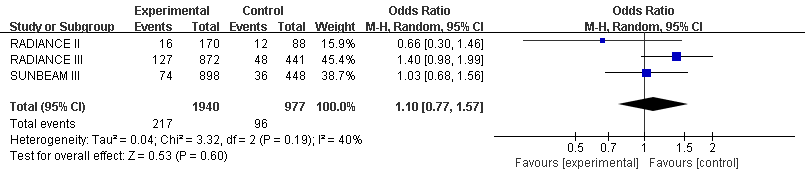


Figure 6. Forest plot of treatment related urinarytract infection ozanimod versus control; CI, confidence interval


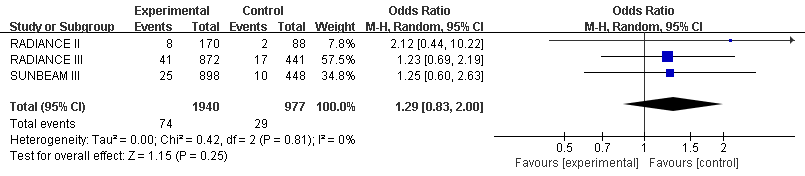


|  |
| --- |

Figure 7. Forest plot of annualized relapse rate during the treatment period 0.5mg ozanimod versus 1.0mg ozanimod; CI, confidence interval


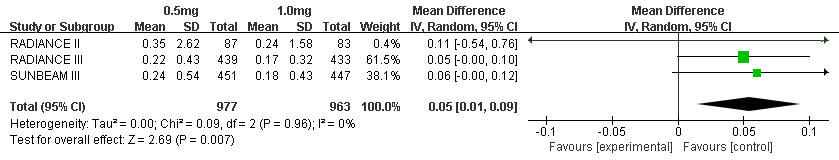


Figure 8. Forest plot of the number of gadolinium-enhancing lesions at the end of the trial 0.5mg ozanimod versus 1.0mg ozanimod; CI, confidence interval


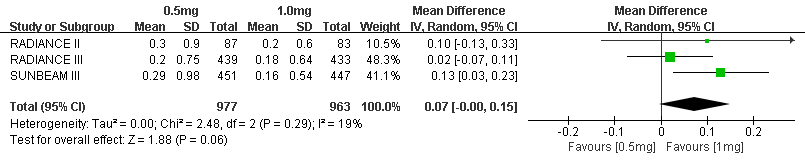


Figure 9. Forest plot of the number of new or enlarging T2 lesions over the treatment period 0.5mg ozanimod versus 1.0mg ozanimod; CI, confidence interval


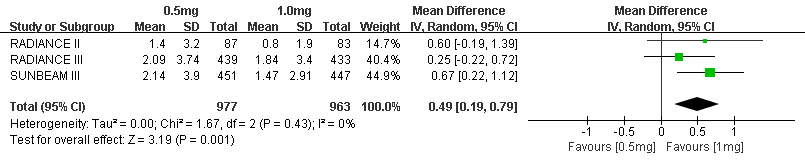


Figure 10. Forest plot of the treatment related adverse events 0.5mg ozanimod versus 1.0mg ozanimod; CI, confidence interval


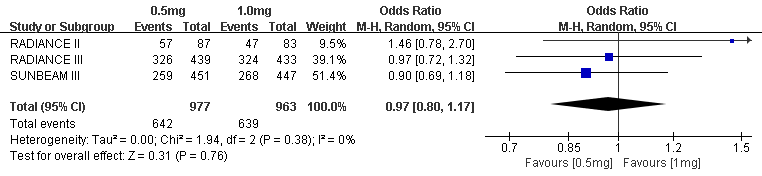


Figure 11. Forest plot of the treatment related nasopharyngitis events 0.5mg ozanimod versus 1.0mg ozanimod; CI, confidence interval


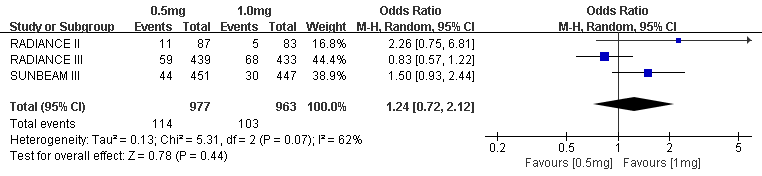


|  |
| --- |
|  |

Figure 11. Forest plot of the treatment related urinarytract infection events 0.5mg ozanimod versus 1.0mg ozanimod; CI, confidence interval


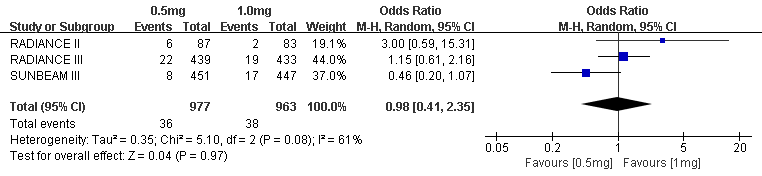

Supplement: Supplementary file 1 [file datasheet1.docx]
